# Interns' knowledge of clinical pharmacology and therapeutics after undergraduate and on-going internship training in Nigeria: a pilot study

**DOI:** 10.1186/1472-6920-9-50

**Published:** 2009-07-28

**Authors:** Kazeem A Oshikoya, Idowu O Senbanjo, Olufemi O Amole

**Affiliations:** 1 Pharmacology Department, Lagos State University College of Medicine, P.M.B 21266, Ikeja, Lagos, Nigeria; 2 Paediatric Department, Lagos State University Teaching Hospital, Ikeja, Lagos, Nigeria

## Abstract

**Background:**

A sound knowledge of pathophysiology of a disease and clinical pharmacology and therapeutics (CPT) of a drug is required for safe and rational prescribing. The aim of this study was therefore to assess how adequately the undergraduate CPT teaching had prepared interns in Nigeria for safe and rational prescribing and retrospectively, to know how they wanted the undergraduate curriculum to be modified so as to improve appropriate prescribing. The effect of internship training on the prescribing ability of the interns was also sought.

**Methods:**

A total of 100 interns were randomly selected from the Lagos State University Teaching Hospital (LASUTH), Ikeja; Lagos University Teaching Hospital (LUTH), Idiaraba; General Hospital Lagos (GHL); the EKO Hospital, Ikeja; and Havana Specialist Hospital, Surulere. A structured questionnaire was the instrument of study. The questionnaire sought information about the demographics of the interns, their undergraduate CPT teaching, experience of adverse drug reactions (ADRs) and drug interactions since starting work, confidence in drug usage and, in retrospect; any perceived deficiencies in their undergraduate CPT teaching.

**Results:**

The response rate was 81%. All the respondents graduated from universities in Nigeria. The ability of the interns to prescribe rationally (66, 81.4%) and safely (47, 58%) was provided by undergraduate CPT teaching. Forty two (51.8%) respondents had problems with prescription writing. The interns would likely prescribe antibiotics (71, 87.6%), nonsteroidal analgesics (66, 81.4%), diuretics (55, 67.9%), sedatives (52, 62.9%), and insulin and oral hypoglycaemics (43, 53%) with confidence and unsupervised. The higher the numbers of clinical rotations done, the more confident were the respondents to prescribe unsupervised (χ^2 ^= 19.98, *P *< 0.001). Similarly, respondents who had rotated through the four major clinical rotations and at least a special posting (χ^2 ^= 11.57, *P *< 0.001) or four major clinical rotations only (χ^2 ^= 11.25, *P *< 0.001) were significantly more confident to prescribe drugs unsupervised.

**Conclusion:**

Undergraduate CPT teaching in Nigeria appears to be deficient. Principles of rational prescribing, drug dose calculation in children and pharmacovigilance should be the focus of undergraduate CPT teaching and should be taught both theoretically and practically. Medical students and interns should be periodically assessed on prescribing knowledge and skills during their training as a means of minimizing prescribing errors.

## Background

The process of admission into medical schools and the medical education curriculum vary from one country to another. Medical students are admitted into Nigerian universities through either the university matriculation examination (UME) or direct entry; having passed physics, chemistry and biology in advanced level GCE or having completed a first degree in any field of science. The University Matriculation Board is responsible for the admission processes. While the students admitted through UME spend a minimum of six years to study medicine, those who were admitted through direct entry spend a minimum of five years. After a year (two semesters) of preliminary study of advanced physics, chemistry, biology and mathematics by the students admitted through the UME, they proceed, along with the direct entry students, to the medical school.

The actual medical training begins in the preclinical years when the students are taught basic medical science subjects which include gross anatomy, embryology, histology, biochemistry and physiology (in the first year); medical microbiology, chemical pathology, haematology and blood transfusion, morbid anatomy, and pharmacology (in the second year and the first half of third year). Medical statistics and ethics, community medicine and anaesthesia are taught in the later part of the third and fifth year. The clinical training spans through the fourth and fifth years. This is the period when the students are taught theoretical and clerkship medicine, obstetrics and gynaecology, surgery, paediatrics and psychiatry.

The Medical and Dental Council of Nigeria (MDCN) is responsible for the design and regulation of undergraduate medical education in Nigeria [1]. The council has recommended that undergraduate pharmacology course should include topics in basic and clinical pharmacology, as well as therapeutics.

In spite of the pitfalls in traditional teaching of pharmacology [2,3], it remains the only method of teaching pharmacology in Nigeria [4]. The traditional teaching is in the form of didactic lectures and bench work practicals. The teaching method often leaves the students to memorize drug information [2-4] and poorly prepare them to prescribe rationally [3,5]. Clinical pharmacology and therapeutics (CPT), the speciality responsible for training doctors in the safe, rational and efficacious use of drugs, has been progressively integrated into the undergraduate curriculum in the USA [6], United Kingdom [7], Netherlands [8], India [9] and Nepal [10] as a way of improving the prescribing knowledge and skills of junior doctors. The integration involved teaching pharmacology in the preclinical year and all through the clinical years in organ system-based manner. This teaching method has focused less on didactic lecture and more on knowledge and skill acquisition on rational drug use. The benefits of the integration system have been widely reported [11,12] and the method has been recommended by the World Health Organization (WHO) as a core intervention to promote rational drug use [13]. Such integration has been advocated in some medical schools in Nigeria [4].

Internship is a period of medical apprenticeship under the supervision of a consultant. The intern is expected to learn clinical skills, perform some clinical procedures and demonstrate a good clinical judgement to arrive at patient management decision. Interns are therefore the most junior doctors in a tertiary hospital. They have been found responsible for a significant number of prescribing errors [14-16]. Globally, prescribing-related errors are common [17-19] and have resulted in a significant patient morbidity and mortality [20-22]. Many concerns have been raised in the United Kingdom [16,23] about the adequacy of undergraduate CPT education in preparing new doctors for the complex task of rational and safe prescribing. Looking into the fact that the majority of prescription-related errors in hospital environment are made by junior doctors [15,24], there is a need to educate the interns and develop an intervention that will improve their prescription qualities. Many studies have evaluated the teaching of undergraduate CPT and its impact on the prescribing ability of junior doctors [25-27]. However, such evaluation has not been done in Nigeria and other African countries.

Traditionally, all newly graduated doctors in Nigeria are required to undergo internship in accredited hospitals for a year before fully registered to practice. The accredited hospitals are listed in the handbook of guidelines on registration as a medical or dental practitioner in Nigeria [28]. The MDCN has recommended that interns should rotate a period of two and a half month through each of medicine, surgery, obstetrics and gynaecology, and paediatrics departments during internship. A month special posting is required in any of radio-diagnosis, radiotherapy, medical microbiology, chemical pathology, haematology, dermatology, psychiatry, morbid anatomy, ophthalmology, orthopaedic or Ear Nose and Throat surgery. The first experience of unsupervised prescribing by the interns begins during the internship. In spite of the wide gap between the periods CPT was taught in medical schools and the commencement of internship, pre-internship CPT was neither taught during employment orientation nor was opportunity provided for continuous medical education (CME) to enable the interns update their knowledge of rational drug use.

This study was therefore aimed to determine how adequately the undergraduate CPT teaching has prepared interns in Nigeria for safe and rational prescribing, and how in retrospect the interns would modify their undergraduate training to improve patient safety when prescribing. The influence of internship training on the prescribing ability of the interns was also sought.

## Methods

A structured questionnaire (Additional file 1), modified from the work of Tobaiqy *et al *[27], was the instrument of study. The questionnaire sought information from the interns about their demographics, undergraduate training in CPT, experience of ADRs and drug interactions since starting work, confidence in drug usage and in retrospects any perceived deficiencies in their undergraduate CPT training. Prescribing errors and polypharmacy are among the leading causes of ADRs in Nigeria [20]. This problem, coupled with poor perception of ADRs reporting by doctors in Nigeria [29], prompted our inclusion of interns' experience with ADRs in the parameters assessed. Most of the questions were leading and required either a yes or no response. The perceived deficiencies in undergraduate CPT teaching were specifically assessed by providing a blank space for the interns to express their views. The questionnaire was initially piloted at the University College Hospital (UCH), Ibadan, which was excluded from the study.

Nigeria is made up of 36 states and the Federal Capital Territory (FCT), Abuja. Twenty one medical schools are established in 16 states and the FCT. Two of the 21 medical schools are in Lagos. All but one medical school is funded by the government. Lagos, the former capital of Nigeria, is the smallest state but the most populous city in Nigeria with an estimated population of about 15 million inhabitants as of 1991 national census. The accredited hospitals for internship in Nigeria include all the affiliated teaching hospitals to the medical schools, military hospitals, a few general hospitals and some private hospitals. The study being a pilot type was conducted in Lagos since it has the largest number of the accredited hospitals for internship.

A total of 100 interns currently doing internship were randomly selected from 5 accredited hospitals, namely the Lagos State University Teaching Hospital (LASUTH), Ikeja; Lagos University Teaching Hospital (LUTH), Idiaraba; General Hospital Lagos (GHL); the EKO Hospital, Ikeja; and Havana Specialist Hospital, Surulere. LASUTH and LUTH; being affiliated hospitals to two of the medical schools and funded by the state and federal governments, respectively, have the capacity to employ more interns than the other hospitals.

The interns who were willing to voluntarily participate in the study were given the questionnaire to fill anonymously. Confidentiality of the information tendered was assured to the participants. The ethics committee of the respective hospitals approved the study.

The data obtained was analysed with SPSS version 13. The relationship between the confidence and experience of the interns to prescribe and the number of clinical rotations done were compared using Chi-square, at a significance level of *P *< 0.05

## Results

### Demographics

Eighty one questionnaires were duly filled and returned, giving a response rate of 81%. A majority of the respondents (48, 59.2%) were between 26–30 years. Only 14 (17.3%) respondents were above 31 years. The respondents were predominantly males (48, 58.8%) and graduated from 13 medical schools. They were predominantly graduates of University of Lagos (33, 40.7%), Lagos State University (16, 19.7%) and University of Ibadan (10, 12.3%). Sixteen (19.7%) respondents had rotated through the four major clinical postings (medicine, paediatrics, surgery, and obstetrics and gynaecology) and a special posting, while 15 (18.5%) had rotated through the four major clinical postings only. Overall, the respondents had rotated through paediatrics (59, 72.8%), obstetrics and gynaecology (55, 67.9%), surgery (54, 66.6%), and medicine (51, 62.9%). Twenty six (32%) respondents had done special postings.

### Undergraduate CPT

Six respondents rated their knowledge of CPT as poor, two rated it as excellent and 20 (24.2%) as good. The remainder (53, 65.4%) rated their knowledge as average. The respondents (66, 81.4% and 47, 58%), were of the opinion that undergraduate CPT teaching had sufficiently equipped them to prescribe rationally and safely, respectively. 42 (51.8%) respondents had problems with prescription writing, 52 (64.2%) had problems with memorizing drug dosage for different age groups. Difficulty in obtaining drug information was a problem of 10 (11.9%) respondents. Difficulty in writing proper prescription (8), identifying drugs with potentials for drug-drug interactions (4), knowing the appropriate drug to use for common clinical conditions (2), identifying drugs by their generic names (2%), and drug dose calculation in paediatric age groups (2%) were other problems identified by the respondents. Among the respondents who perceived that the undergraduate CPT teaching had prepared them to prescribe rationally, inexperience (55, 67.9%), lack of confidence (21, 25.9%) and lack of supervision and advice from their senior colleagues (22, 27.1%) were the key factors that may affect their ability to prescribe appropriately.

### Internship training

To assess influence of internship training on the prescribing skills of the respondents, they were asked to rate their level of confidence when prescribing drugs unsupervised from a specific list of drugs, or when prescribing to a special group of patients such as children, the elderly and pregnant women. Table 1 shows the list of drugs that are likely to be prescribed with confidence. A high number of the respondents would confidently prescribe vitamins and minerals, antimalarials, antacids and anti-ulcer drugs which have low risk of adverse reactions. Similarly, a low number of the respondents would confidently prescribe immunosuppressant and cytotoxic drugs which have high risk for adverse reactions. However, of great concern was the high number of the respondents who would confidently prescribe (antibiotics, NSAIDs, diuretics, sedatives and insulin/oral hypoglycaemics) drugs with high potentials for adverse reactions without supervision. Table 2, on the other hand, shows that a majority of the respondents were not confident to prescribe many drugs to a special group of patients. There was a significant relationship between the perceived levels of confidence, experience and the number of clinical postings the respondents had rotated through. The higher the numbers of clinical rotations done, the more confident were the respondents to prescribe without supervision (χ^2 ^= 19.98, *P *< 0.001). However, there was no significant difference in the number of clinical rotations and the experience of the respondents at prescribing drugs unsupervised (χ^2 ^= 2.22, *P *= 0.891). The respondents who had rotated through the four major clinical postings and a special posting (χ^2 ^= 11.57, *P *< 0.001) or four major postings only (χ^2 ^= 11.25, *P *< 0.001) were significantly more confident to prescribe drugs without supervision. Similarly, respondents who had rotated through only one major posting were significantly not confident to prescribe drugs without supervision (χ^2 ^= 4.17, *P *= 0.039).

**Table 1:** List of drugs that interns would comfortably prescribe without supervision

**Drugs**	**Frequency per total respondents (n = 81)**	**Percentage** **(%)**
Antimalarials	78	96.2
Vitamins and minerals	72	88.8
Antibiotics	71	87.6
NSAIDs	66	81.4
Antacids and anti-ulcer	62	76.5
Diuretics	55	67.9
Antihistamines	55	67.9
Laxatives	54	66.6
Anti-asthma inhaler	53	65.4
Sedatives	51	62.9
Antiemetics	50	61.7
Insulin and oral hypoglycaemics	43	53.0
Vaccines	42	51.8
Anticonvulsants	39	48.1
Aminophylline	39	48.1
Steroids	28	34.5
Opiate analgesics	27	33.3
Digoxin	17	20.9
Antidepressants	16	19.7
Antipsychotics	15	18.5
Anti-Parkinsons'	7	8.6
Immunosupressants	7	8.6
Cytotoxics	6	7.4

NSAIDs: Non-steroidal anti-inflammatory drugs

**Table 2:** List of drugs that interns would reluctantly prescribe to children, elderly, pregnant women and patient with renal and/or liver impairment without supervision

**Drugs**	**Frequency per total respondents (n = 81)**	**Percentage** **(%)**
Cytotoxics	70	86.4
Digoxin	62	76.5
Immunosupressants	60	74.0
Antipsychotics	58	71.6
Anti-Parkinsons'	57	70.3
Antidepressants	55	67.9
Steroids	42	51.8
Anticonvulsants	34	41.9
Aminophylline	33	40.7
Opiate analgesics	32	39.5
Vaccines	28	34.5
Insulin and oral hypoglycaemics	27	33.3
Diuretics	21	25.9
Sedatives	20	24.6
Anti-asthma inhaler	15	18.5
Laxatives	15	18.5
Antihistamines	12	14.8
Antibiotics	11	13.5
Antiemetics	9	11.1
Antacids and anti-ulcer	9	11.1
Vitamins and minerals	6	7.4
NSAIDs	5	6.1
Antimalarials	3	3.7

NSAIDs: Non-steroidal anti-inflammatory drugs

The respondents considered safety (59, 72.8%), efficacy (24, 29.6%) and cost (14, 17.2%) of the drugs in their order of importance when prescribing.

### Adverse drug reactions (ADRs)

54 (66.6%) respondents had witnessed an ADR during the internship period. The ADR resulted in hospitalization (24, 44.4%), prolonged hospital stay (21, 38.8%), morbidity (11, 20.3%) and death (5). Drug-drug interactions accounted for six of the witnessed ADRs. Less than half (42.5%) of the respondents thought that the witnessed ADRs were predictable and 41 (75.9%) cases were avoidable. Unfortunately, a majority of the witnessed ADRs (44, 81.4%) went unreported, with only 10 (18.5%) respondents reporting to the appropriate authority within the hospitals and four respondents reporting to the National Pharmacovigilance Centre (NPC). Many of the respondents (32, 59.2%) felt a proper training in CPT would have prevented the witnessed ADRs and almost half of the respondents (58%) had not been sufficiently taught how to prevent ADRs. Topics related to ADRs were discussed with less than half of the respondents during the internship.

### Sources of reference for drug prescribing

**Figure 1:**
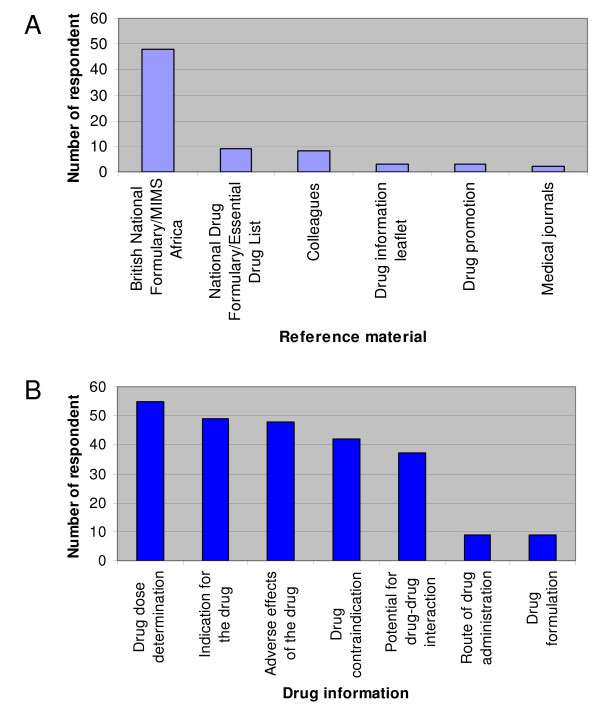
**The materials used for reference and the drug information sought from them are shown in parts A and B respectively**.

A majority of the respondents (70, 86.5%) routinely checked a reference source before prescribing drugs. The materials used for reference and the drug information sought from them are shown in Figures 1A and 1B, respectively.

### Possible areas of improving teaching in CPT and prescribing

**Figure 2:**
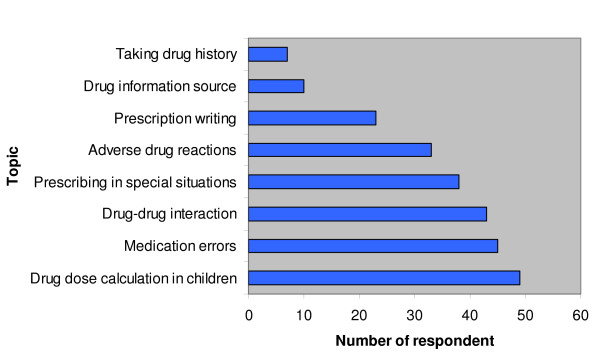
**Suggested topics by the interns that should be well taught in undergraduate training**.

Retrospectively, the interns were asked to suggest topics in CPT that require wider coverage. Figure 2 shows that drug dose calculation in children (49, 60.8%) and medication errors (45, 55.9%) were the two most frequently suggested topics. However, drug information source (10) and how to take drug history (7) were the least suggested topics.

Modifications of the undergraduate training, as perceived by the respondents, include problem based learning of CPT (44, 54%), bed-side teaching of pharmacology (42, 52.5%), orientation lectures in CPT before the commencement of internship (41, 50.8%) and seminars and tutorials in CPT (33, 40.3%).

## Discussion

The majority of the respondents rated their knowledge of undergraduate CPT as average and good, thus indicating that undergraduate CPT teaching in Nigeria was likely good. This may probably explain the high number of respondents who perceived themselves sufficiently prepared to prescribe rationally. However, these findings did not correlate with the high proportion of respondents who had problems with prescription writing and those who memorized drug dosage for different age groups. Previous studies have shown that the prescribing knowledge of final year medical students in Nigeria did not correlate with their prescribing skills [5]. Only the theoretical knowledge of CPT is imparted when medical students are taught. Practical prescribing and regular assessment of the prescribing skills were rarely practised during undergraduate CPT teaching [5,25,27]. Medical students are not formally taught paediatric drug dose calculation either in pharmacology or paediatrics; they have demonstrated difficulty in calculating paediatric drug doses and are willing to learn if formally taught [30,31]. This may explain why quite a number of the respondents would memorize drug dosages. Knowledge acquired by memorizing is usually brief; therefore interns are likely to increase medication errors in Nigeria.

Internship is a period of medical apprenticeship under the supervision of senior doctors, especially the consultants. During the training, interns picked up some prescribing knowledge and skills, through informal CPT teaching by the medical officers, residents and consultants, which might have improved their experience and confidence to prescribe appropriately. A majority of the respondents were more confident to prescribe a variety of drugs unsupervised. However, their chosen drugs tended to reflect the pattern of ailment they have attended to and their routine prescribing workload, rather than on their knowledge of potential adverse effects of the drugs. The less concern shown by the respondents to the chosen drugs is reflected in the large numbers who felt confident to prescribe antibiotics, nonsteroidal analgesics, diuretics, sedatives, and insulin and oral hypoglycaemics unsupervised. These are drugs mostly involved in severe and fatal ADRs [19,32,33]. Lack of prudence and caution in prescribing these drugs is inappropriate and suggests that prescribing confidence results from prolong practice, rather than guidance about the true risks of the drugs and the complexities involved.

Most of the respondents were not confident to prescribe to children, elderly, pregnant women and people with impaired renal or liver functions (Table 2). Therefore medication use in these special people should remain a focus of undergraduate CPT teaching. Confident prescribing in this study was significantly dependent on the internship exposures. The more the number of clinical rotations, the more confident are the respondents at prescribing unsupervised but contrarily, the number of clinical rotations did not significantly contribute to their prescribing experience. The confidence of the respondents might have come with practice, responsibility, familiarity with frequently used drugs on the ward and adequate supervision by their senior colleagues [34].

About two-third of the respondents claimed they had witnessed ADRs during the internship, a small proportion of which was due to drug-drug interaction. A significant number of the ADRs resulted in prolonged hospitalisation, morbidity and mortality. Similar results had been reported among paediatric age groups in Nigeria [20] and other studies from developed countries [32,33]. The fact that 75.9% of the ADRs were avoidable, 40% being predictable and over half of the respondents not being taught how to prevent ADRs, would necessitate a focus on the preventive measures of ADRs in undergraduate CPT teaching. A majority of the witnessed ADRs that went unreported and a significantly low percentage of the cases reported to the National Pharmacovigilance Centre strongly suggest a poor ADRs monitoring system in Nigeria. Therefore more awareness on ADRs reporting and surveillance needs to be created among doctors in Nigeria.

Reference to drug formularies for prescribing information has been reported as an important step to preventing ADRs [35]. It is highly commendable that this was practised by a majority of the respondents. However, making a little reference to the Nigerian national drug formulary/essential drug lists is of great concern. The implication of this is that the respondents are likely to prescribe drugs that are more expensive and unavailable locally at all time; this totally negates the WHO's guidelines for rational prescribing [36]. Deficiencies in the knowledge and basic skills of prescribing, as well as deficiency in taking a good drug history, are responsible for a significant number of medication errors [37,38]. These topics were however given a low priority in undergraduate CPT teaching by a majority of the respondents, which further shows, that complacency of the respondents towards achieving appropriate prescribing skills, may be very difficult to change. It is also surprising that alternatives to teaching improvement of CPT were not suggested by any of the respondents. The use of a prescribing checklist as an aide-memoire has been suggested by Jackson et al [39] as a means of improving prescribing practice. This can be developed for medical students for their familiarisation and use during internship.

Considering the large number of medical schools in Nigeria, the proportion of interns studied is relatively small, therefore the results cannot necessarily be generalised as the views of all the interns who were medically trained in Nigeria. This is a pilot study and has given us an insight into undergraduate CPT teaching in Nigeria. It has also identified deficiencies in CPTS teaching; similar to those of other smaller studies in the UK [26,27], which need to be addressed. The study relies on self-rated confidence rather than objective demonstration of prescribing knowledge and skills. Confidence of the interns to prescribe rationally may not necessarily translate into good prescribing. While the supposedly confident interns may be incompetent to prescribe appropriately, the insecure ones may prescribe better. Studies have shown that many doctors who were able to prescribe confidently were actually unable to write clear and legible prescriptions for pharmacists to dispense or nurses to administer without confusion [40,41].

The diverse nature of the respondents with varying clinical experience and internship activities were likely to influence their perceptions of the undergraduate CPT teaching and some of their responses may not be genuinely true. Like other studies [25-27], there was an overlap between the undergraduate CPT teaching and postgraduate training/employment. The overlap is inevitable in studies that assess the prescribing knowledge of junior doctors because principle of proper prescribing is relevant to both medical students and practising doctors [33].

This study was biased towards respondents from two medical schools (Lagos State University College of Medicine and College of Medicine, University of Lagos) because most of the respondents were employed by the teaching hospitals affiliated to their medical schools. A large study that would include interns from all the teaching hospitals in Nigeria would likely eliminate this bias and give better views of interns about CPT teaching in Nigeria.

## Conclusion

In spite of a number of methodological limitations, this pilot study has shown that undergraduate CPT teaching in Nigeria is inadequate. Interns would like principles of rational prescribing; paediatric drug dose calculation; and ADRs prevention, monitoring and reporting to form part of the core curriculum of undergraduate CPT teaching in Nigeria. Theoretical and practical CPT teaching coupled with frequent assessment of the knowledge and skills acquired by the students, would likely improve their rational drug use as interns. Internship training appears to increase the prescribing confidence of interns which need to be assessed objectively during and after internship.

## Abbreviations

LASUTH: Lagos State University Teaching Hospital; LUTH: Lagos University Teaching Hospital; GHL: General Hospital Lagos; CPT: Clinical Pharmacology and Therapeutics; ADR: Adverse Drug Reaction; G.C.E: General Certificate Examination; MDCN: Medical and Dental Council of Nigeria; FCT: Federal Capital Territory; WHO: World Health Organization; and NPC: National Pharmacovigilance Centre.

## Competing interests

The authors declare that they have no competing interests.

## Authors' contributions

KAO conceived the study, designed the study and questionnaire, reviewed the statistical analysis, and drafted the manuscript. IOS performed the statistical analysis, and participated in drafting the manuscript. OOA participated in the design of the questionnaire, performed the data entry and critically reviewed the manuscript. All the authors read and approved the final manuscript.

## Pre-publication history

The pre-publication history for this paper can be accessed here:



## Supplementary Material

Additional file 1 **Questionnaire for evaluating Interns' knowledge of clinical pharmacology and therapeutics**. The questionnaire sought information about the demographics of the interns, their undergraduate CPT teaching, experience of adverse drug reactions (ADRs) and drug interactions since starting work, confidence in drug usage and, in retrospect; any perceived deficiencies in their undergraduate CPT teaching. Click here for file
