# Supplementary material for: Interns' knowledge of clinical pharmacology and therapeutics after undergraduate and on-going internship training in Nigeria: a pilot study
Source: BMC Med Educ. 2009 Jul 28;9:50. doi: 10.1186/1472-6920-9-50 (PMC2724475; doi:10.1186/1472-6920-9-50)
Supplement: Additional file 1 — Questionnaire for evaluating Interns' knowledge of clinical pharmacology and therapeutics. The questionnaire sought information about the demographics of the interns, their undergraduate CPT teaching, experience of adverse drug reactions (ADRs) and drug interactions since starting work, confidence in drug usage and, in retrospect; any perceived deficiencies in their undergraduate CPT teaching. [file 1472-6920-9-50-S1.doc]

**Interns’ knowledge of clinical pharmacology and therapeutics**

This is a self-sponsored study is aimed at assessing knowledge of clinical pharmacology and therapeutics (CPT) of medical graduates from Nigerian universities. Based on our findings, we hope to recommend to the MDCN the possible ways of improving undergraduate CPT teaching.

Please select the appropriate options and fill-in the blank spaces provided. Ensure all the questions are answered. Filling-in this questionnaire is taken as your consent to participate. All the information tendered would be treated with utmost confidentiality.

**Demographics**

1. How old are you?

(a) ≤ 20 years (b) 21-25 years (c) 26-30 years (d) 31-35 years (e) ≥ 35 years.

1. What is your gender?

(a) male (b) female.

1. Where did you do your undergraduate medical training?

(a) Nigeria (b) abroad

1. If trained in Nigeria, please name the institution? ……………………………..
2. In which year did you graduate? (Please specify)………………………………
3. Which of the following clinical rotations have you done since you started internship (including your current posting)?

(a) medicine (b) surgery (c) obstetrics and gynaecology (d) paediatrics (e) radiodiagnosis (f) radiotherapy (g) medical microbiology (h) chemical pathology (i) haematology (j) dermatology (k) psychiatry (l) morbid anatomy (m) ophthalmology (n) orthopaedic (o) Ear Nose and Throat surgery

**Undergraduate CPT teaching**

1. Were you taught CPT in your undergraduate pharmacology?

(a) yes (b) no

1. How would you rate your knowledge as at graduation?

(a) very poor (b) poor (c) average (d) good (e) excellent

1. Do you feel that your undergraduate training has prepared you to prescribe safely? (a) yes (b) no
2. Do you feel that your undergraduate training has prepared you to prescribe rationally?

(a) yes (b) no

1. If the above is yes, what other factors that may affect your ability to prescribe rationally? (Please specify)……………………………………………
2. Have you had any specific problems with prescribing during your internship training?

(a) yes (b) no

1. If the above is yes, what are the specific problems? (Please specify)……………………………………………………………………………...

**Internship training**

1. Which of the following drugs would you comfortably prescribe without supervision? (a) antimalarials (b)vitamins and minerals (c) antibiotics (d) NSAIDs (e) antacids and anti-ulcer (f) diuretics (g) antihistamines (h) laxatives (i) anti-asthma inhaler (j) sedatives (k) antiemetics (l) insulin and oral hypoglycaemics (m) vaccines (n) anticonvulsants (o) aminophylline (p) steroids (q) oipiate (r) analgesics (s) digoxin (t) antidepressants (u) antipsychotics (w) anti-Parkinsons’(x) immunosupressants (y) cytotoxics
2. Which of the following drugs would you reluctantly prescribe to the elderly, children, pregnant women or people with renal /liver impairement? (a) antimalarials (b)vitamins and minerals (c) antibiotics (d) NSAIDs (e) antacids and anti-ulcer (f) diuretics (g) antihistamines (h) laxatives (i) anti-asthma inhaler (j) sedatives (k) antiemetics (l) insulin and oral hypoglycaemics (m) vaccines (n) anticonvulsants (o) aminophylline (p) steroids (q) oipiate (r) analgesics (s) digoxin (t) antidepressants (u) antipsychotics (w) anti-Parkinsons’(x) immunosupressants (y) cytotoxics
3. What are the most important factors you would consider when prescribing a drug? (a) safety (b) efficacy (c) cost (d) others (please specify)………………………

**Knowledge of adverse drug reactions (ADRs)**

1. Do you understand the term ADRs?

(a) yes (b) no

1. Have you witnessed any ADRs since you started internship?

(a) yes (b) no

1. The likely cause of the ADRs is?

(a) drug-drug interaction (b) medication error (c) idiosyncratic reaction (d) others (please specify)………………………………………

1. If the above is yes, which of the following did it result in?

(a) short hospitalisation (b) prolonged hospitalisation (c) morbidity (d) death

1. Do you consider the ADRs avoidable?

(a) yes (b) no

1. Do you consider the ADRs predictable?

(a) yes (b) no

1. Do you think a good knowledge of undergraduate CPT teaching would have prevented the ADRs?

(a) yes (b) no

1. Did you report the ADRs?

(a) yes (b) no

1. If the above is yes, whom did you report to?

(a) ADRs monitoring committee of the hospital (b) National Pharmacovigilance centre (c) head of department (d) others (please specify)…………………………

1. Were you taught how to prevent ADRs occurrence in your undergraduate CPT teaching?

(a) yes (b) no

1. Has anybody ever discussed ADRs related topics with you since you started internship training?

(a) yes (b) no

**Drug information references and how to improve undergraduate CPT teaching**

1. Do you routinely check information about drugs before prescribing?

(a) yes (b) no

1. If the above is yes, which materials do you consult? (Please specify) …………………………………………………………………………….
2. Retrospectively, do you think undergraduate CPT teaching should be improved? (a) yes (b) no
3. If the above is yes, please suggest ways of improving the teaching (please specify)……………………………………………………………………………
4. Please list the topics in CPT that you consider important to receive much teaching focus and coverage (please specify)……………………………………………
